# Traffic-driven epidemic outbreak on complex networks: How long does it take?

**DOI:** 10.1063/1.4772967

**Published:** 2012-12-28

**Authors:** Han-Xin Yang, Wen-Xu Wang, Ying-Cheng Lai

**Affiliations:** 1 Department of Physics, Fuzhou University, Fuzhou 350108, China; 2 Department of Systems Science, School of Management and Center for Complexity Research, Beijing Normal University, Beijing 100875, China; 3 School of Electrical, Computer and Energy Engineering, Arizona State University, Tempe, Arizona 85287, USA

## Abstract

Recent studies have suggested the necessity to incorporate traffic dynamics into the process of epidemic spreading on complex networks, as the former provides support for the latter in many real-world situations. While there are results on the asymptotic scope of the spreading dynamics, the issue of how fast an epidemic outbreak can occur remains outstanding. We observe numerically that the density of the infected nodes exhibits an exponential increase with time initially, rendering definable a characteristic time for the outbreak. We then derive a formula for scale-free networks, which relates this time to parameters characterizing the traffic dynamics and the network structure such as packet-generation rate and betweenness distribution. The validity of the formula is tested numerically. Our study indicates that increasing the average degree and/or inducing traffic congestion can slow down the spreading process significantly.

A spreading process cannot occur on a complex network without a backbone traffic dynamics that transports certain physical quantity across the network. For example, a computer virus may become widespread through email exchanges, and a disease/virus can spread globally through air transportation. A complete understanding of epidemic spreading dynamics thus requires incorporation of some kind of traffic dynamics into the spreading process. This has been a topic of several recent studies, where the focus has been on the asymptotic extent of the spreading dynamics, e.g., the fraction of infected nodes after the process terminates. The aim of our work is to address the issue of timing associated with traffic-driven epidemic spreading dynamics. In particular, we incorporate a shortest-path-based type of traffic dynamics into the standard two-state epidemic spreading model. We find that the density of the infected nodes exhibits an exponential increase with time in the early stage, rendering definable a characteristic time for the spreading process. Numerical results and theoretical reasoning indicate that this time depends on various parameters characterizing the traffic dynamics and network structure, such as packet-generation rate, spreading rate, and betweenness distribution of the network. For example, large-scale outbreaks occur more quickly as the packet-generation rate and the spreading rate increase. A somewhat counterintuitive finding is that an increase in the average connectivity tends to slow down the spreading process. Our study of how epidemic propagates among nodes of different degrees indicates that large-degree nodes are infected first, followed by a progressive spreading over small-degree nodes. In addition, we find that the emergence of traffic congestion can slow down the epidemic outbreak significantly, providing insights into developing effective strategies to prevent large-scale epidemic outbreaks on complex networks.

## I. INTRODUCTION

The outbreaks of diseases in the human society or viruses in networked technological systems are an issue of paramount importance. The past decade has witnessed a great deal of effort in understanding the dynamical process of epidemic spreading on complex networks.^1–10^ In most early theoretical models, the propagation of virus from one node to another is tacitly assumed to be a reaction process, that is, an infected node can affect any of the nodes in its neighborhood with a fixed probability at each time step. This picture, however, may be idealized. In many real-world situations, even when there is a link connecting two neighboring nodes, propagation of infection will not occur unless there is a kind of traffic dynamics on the network that can physically transport the virus from one node to another. For example, a computer virus can spread over the Internet but only through some transportation process such as email exchanges. Without such a physical process of data transmission, even if there is a path linking two computers, the virus will not propagate from one computer to another. Potentially fast spreading of infectious diseases among different regions is another example, which can be accelerated tremendously by air travel. To gain a more complete and physical understanding of the dynamical process of epidemic spreading on complex networks, some sort of underlying traffic dynamics must be taken into account.

In a recent work, Meloni *et al.* introduced a modeling approach to this problem by incorporating traffic dynamics in epidemic spreading.^11^ Specifically, they combined the classical susceptible-infected-susceptible (SIS) model^12^ with a class of transport dynamics where contagion is carried and propagated by packets traveling across the network. The probability that the epidemic spreads from infected to susceptible nodes depends on the traffic flow. A susceptible node is more likely to be infected if it receives more packets from infected neighbors. Their main result was that the epidemic threshold tends to decrease as the traffic flow is intensified. Subsequently, the issue was investigated of how local-routing-based traffic dynamics affects epidemic spreading,^13^ with the finding that the spreading dynamics can be suppressed by routing control in the sense that the epidemic threshold can be maximized by optimizing some key parameter characterizing the routing process. In these works, the quantity of interest was the final scope of the epidemics in terms of, for instance, the asymptotic fraction of the infected nodes in the equilibrium state. To our knowledge, the important issue of how long it takes for epidemic outbreak to occur in traffic-driven spreading dynamics has not been considered.

In this paper, we address the issue of outbreak time associated with traffic-driven epidemics on complex networks. We employ a model where the transport of packages is executed along the shortest paths in the network. A characteristic time τ is introduced based on numerical observations, which is the time it takes for a certain large fraction of nodes to be infected. To be concrete, we focus on scale-free networks^14^ and derive a theoretical formula of τ. The formula indicates that, in the absence of traffic congestion, large-scale outbreak can occur faster as the traffic flow is intensified. However, quite counter-intuitively, as the average connectivity of the network is increased, τ becomes larger so that the occurrence of outbreak will be slower. The onset of traffic congestion will decrease the speed of epidemic outbreak.

In Sec. II, we describe the traffic-driven epidemic model. In Sec. III, we present numerical results and a theoretical derivation of the characteristic time τ. A brief conclusion is presented in Sec. IV.

## II. MODEL

In order to study the dynamical evolution of traffic-driven epidemic outbreaks, we consider the standard susceptible-infected (SI) model^12^ of epidemic spreading. In a network of size *N*, at each time step, λN new packets are generated with randomly chosen sources and destinations, and each node can deliver at most *C* packets toward their destinations. Packets are forwarded according to various routing algorithms.^15–19^ To be concrete, we use the shortest-path routing protocol.^20,21^ The queue length of each agent is assumed to be unlimited. The first-in-first-out principle applies to the queue. Each newly generated packet is placed at the end of the queue of its source node. Once a packet reaches its destination, it is removed from the system.

Nodes can be in two discrete states, either susceptible or infected. Initially, we set one randomly selected node to be infected and all other nodes to be susceptible. The infection spreads in the network through packet exchanges. A susceptible node has probability β being infected each time when it receives a packet from an infected neighbor.

## III. RESULTS

We use scale-free networks with the power-law degree distribution:^14^ $P(k)=2m^{2}k^{-3}$, where *m* is the minimum node degree and the average connectivity of the network is 〈k〉=2m. The network size is fixed to be *N* = 2000.

**FIG. 1.**
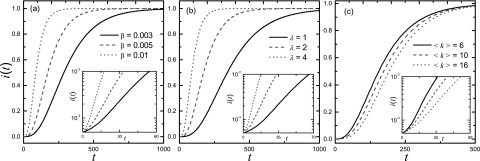
Density of infected nodes *i*(*t*) as a function of time *t* for (a) different values of the spreading rate β, (b) different values of the packet-generation rate λ, and (c) different values of the average connectivity 〈k〉. For (a), λ=2 and 〈k〉=10; For (b), β=0.005 and 〈k〉=10; For (c), β=0.005 and λ=2. The insets show the evolution of *i*(*t*) in the early times.

We first consider the scenario that the node delivery capacity is infinite, C→∞. In this case, traffic congestion does not occur in the network. Let *S*(*t*) and *I*(*t*) be the numbers of susceptible and infected nodes at time *t*, respectively. Figures 1(a)–1(c) show the density of the infected nodes *i*(*t*) = *I*(*t*)/*N* with time *t* for different values of the spreading probability β, the packet-generation rate λ, and the average connectivity 〈k〉, respectively. It can be seen that *i*(*t*) increases more quickly for larger values of β and λ but surprisingly for smaller values of 〈k〉. Considering that, in the absence of traffic dynamics, increasing the average connectivity can accelerate epidemic spreading,^22^ the result in Fig. 1(c) appears to be counterintuitive. The insets of Figs. 1(a)–1(c) indicate an initial exponential increasing phase in the density of the infected nodes with time, from which a characteristic time for the outbreak can be meaningfully defined. In the following, we provide a physical theory to understand how the spreading probability, the packet-generation rate and the average connectivity affect the exponentially growing behavior.

A scale-free network is heterogeneous, rendering necessary consideration of the effect of degree heterogeneity on the dynamical evolution. We define the density of infected nodes of degree *k* as $i_{k}(t)=I_{k}(t)/N_{k}$, where $N_{k}$ and $I_{k}(t)$ are the numbers of nodes and of infected nodes within each degree class *k*, respectively. According to the heterogeneous mean-field theory,^2,11,22^ we can write the reaction-rate equation for the epidemic dynamics as

$$\frac{di_{k}(t)}{\mathrm{dt}}=\beta \lambda Nb_{\mathrm{alg}}^{k}[1-i_{k}(t)]\Theta (t),$$ (1)

where the right-hand side takes into account the probability that a node with *k* links belongs to the susceptible class represented by [$1-i_{k}(t)$] and can become infected via packets traveling from the infected nodes. The latter process is determined by the spreading probability β, the number of packets $\lambda Nb_{\mathrm{alg}}^{k}$ that a node of degree *k* receives at each time step, and the probability Θ(t) that a packet travels through a link pointing to an infected node. In the case of uncorrelated networks, Θ(t) is independent of the degree and is given by^11^

$$\Theta (t)=\frac{\sum_{k}b_{\mathrm{alg}}^{k}P(k)i_{k}(t)}{\sum_{k}b_{\mathrm{alg}}^{k}P(k)},$$ (2)

where $b_{\mathrm{alg}}^{k}$ is the algorithmic betweenness,^23^ which denotes the number of packets passing through a node for a given routing protocol and packet-generation rate λ=1/N. In the case of shortest-path routing, the algorithmic betweenness is equal to the topological betweenness. In the initial spreading stage, terms of order $O(i^{2})$ can be ignored so that Eq. (1) can be simplified as

$$\frac{di_{k}(t)}{\mathrm{dt}}=\beta \lambda Nb_{\mathrm{alg}}^{k}\Theta (t).$$ (3)

Substituting Eq. (2) into Eq. (3), we obtain

$$\frac{d\Theta (t)}{\mathrm{dt}}=\frac{\beta \lambda N〈b_{\mathrm{alg}}^{2}〉\Theta (t)}{〈b_{\mathrm{alg}}〉},$$ (4)

where $〈b_{\mathrm{alg}}〉=\sum_{k}b_{\mathrm{alg}}^{k}P(k)$ and $〈b_{\mathrm{alg}}^{2}〉=\sum_{k}{\left(b_{\mathrm{alg}}^{k}\right)}^{2}P(k)$. From Eq. (4), we obtain

$$\Theta (t)=\Theta (0)e^{1/\tau },$$ (5)

where

$$\tau =\frac{〈b_{\mathrm{alg}}〉}{\beta \lambda N〈b_{\mathrm{alg}}^{2}〉}.$$ (6)

Substituting Eq. (5) into Eq. (3), we get

$$i_{k}(t)=i_{k}(0)+\frac{\Theta (0)b_{\mathrm{alg}}^{k}〈b_{\mathrm{alg}}〉}{〈b_{\mathrm{alg}}^{2}〉}(e^{t/\tau }-1).$$ (7)

Using the uniform initial condition $i_{k}(0)=i(0)$, we can rewrite Eq. (7) as

$$i_{k}(t)=i(0)\left[\frac{b_{\mathrm{alg}}^{k}〈b_{\mathrm{alg}}〉}{〈b_{\mathrm{alg}}^{2}〉}(e^{t/\tau }-1)+1\right].$$ (8)

Substituting Eq. (8) into $i(t)=\Sigma_{k}P(k)i_{k}(t)$, we obtain

$$i(t)=i(0)\left[\frac{{〈b_{\mathrm{alg}}〉}^{2}}{〈b_{\mathrm{alg}}^{2}〉}(e^{t/\tau }-1)+1\right],$$ (9)

which indicates that initially, the density of infected nodes increases exponentially over time. Because of the exponentially growing behavior, a time characterizing the epidemic break can be properly defined, which is τ, where a larger value of τ signifies slower growth. From Eq. (6), one can see that τ is directly proportional to the ratio of the first to the second moment of the algorithmic betweenness distribution and is inversely proportional to the spreading probability and the packet-generation rate. The exponentially growing behavior is true for the small values of $\beta \lambda N〈b_{\mathrm{alg}}〉$, i.e., $\beta \lambda N〈b_{\mathrm{alg}}〉\ll 1$.

**FIG. 2.**
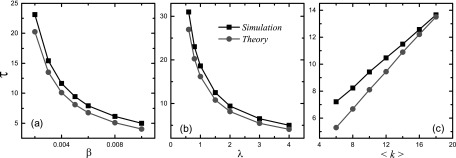
Comparison between numerical and theoretical values of the characteristic time τ: (a) τ versus the spreading probability β for λ=2 and 〈k〉=10, (b) τ versus the packet-generation rate λ for β=0.005 and 〈k〉=10, and (c) τ versus the average connectivity 〈k〉 for β=0.005 and λ=2. The theoretical values are calculated from Eq. (6).

Validity of our physical analysis can be established by comparing the numerically calculated dependence of τ on the spreading probability β, the packet-generation rate λ and the average connectivity 〈k〉 with the theoretical predictions. From Fig. 2, one can see that the theoretical predictions agree with numerical results qualitatively. In Eq. (1), the algorithmic betweenness $b_{\mathrm{alg}}^{k}$ is assumed to be the same for nodes in the same degree class *k*. However, $b_{\mathrm{alg}}^{k}$ actually fluctuates for different nodes with the same degree, leading to deviation between numerical and theoretical values. As the average connectivity 〈k〉 increases, the difference of the algorithmic betweenness among nodes in the same degree class reduces, yielding better agreement between numerical observations and theoretical predictions [see Fig. 2(c)].

An interesting issue is how the epidemic dynamics affects nodes with different degrees. This can be assessed by measuring the average degree of the newly infected nodes as a function of time *t*, which is defined as

$$〈k_{\inf}(t)〉=\frac{\sum_{k}k[I_{k}(t)-I_{k}(t-1)]}{I(t)-I(t-1)}.$$ (10)

**FIG. 3.**
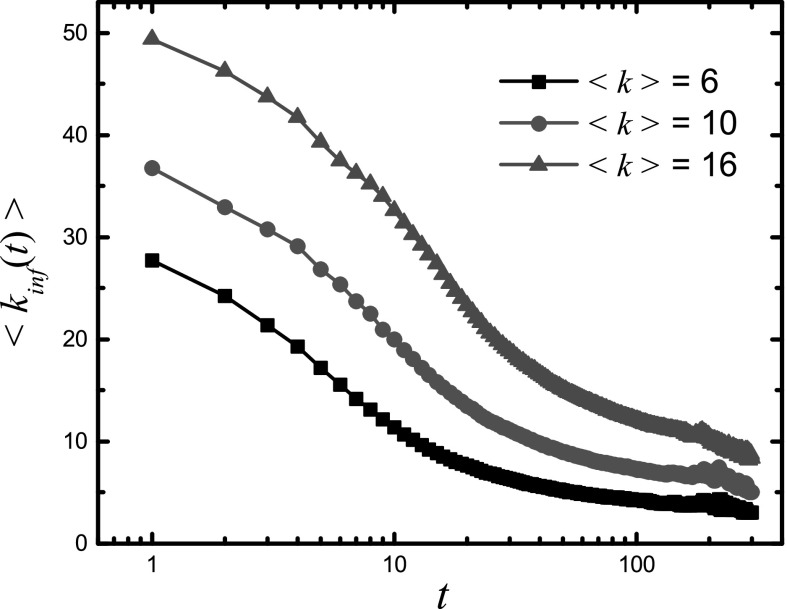
Average degree $〈k_{\inf}(t)〉$ of the newly infected nodes as a function of time for different values of the average connectivity 〈k〉. The spreading and packet-generation rates are β=0.02 and λ=2, respectively.

Figure 3 shows a decreasing behavior of $〈k_{\inf}(t)〉$ with *t*, indicating that the epidemic dynamics first infects the large-degree nodes in the network, and the infection then spreads over the network to progressively smaller-degree nodes in a cascade-like manner. This behavior agrees with intuition, as hub nodes tend to receive far more packets than an average node in a scale-free network, and they are then significantly more likely to be infected first. Packets carrying the infection then spread to nodes with slightly smaller degrees, and so on, until a large fraction of the entire network is infected.

**FIG. 4.**
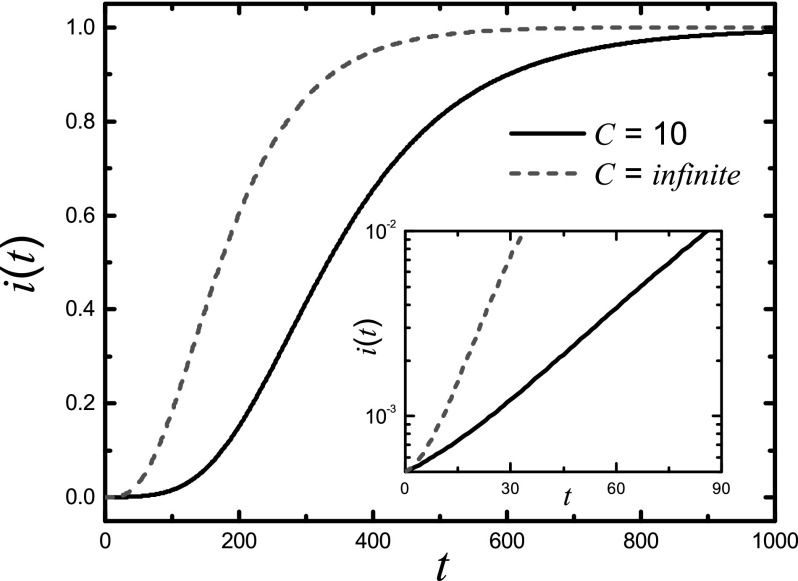
Density of infected nodes *i*(*t*) as a function of time *t* for *C* = 10 and C→∞. The average connectivity is 〈k〉=10, the spreading probability is β=0.01 and the packet-generation rate is λ=1. For *C* = 10, the critical packet-generating rate is $\lambda_{c}≃0.05$. The inset shows the evolution of *i*(*t*) at the initial stage of epidemic spreading.

**FIG. 5.**
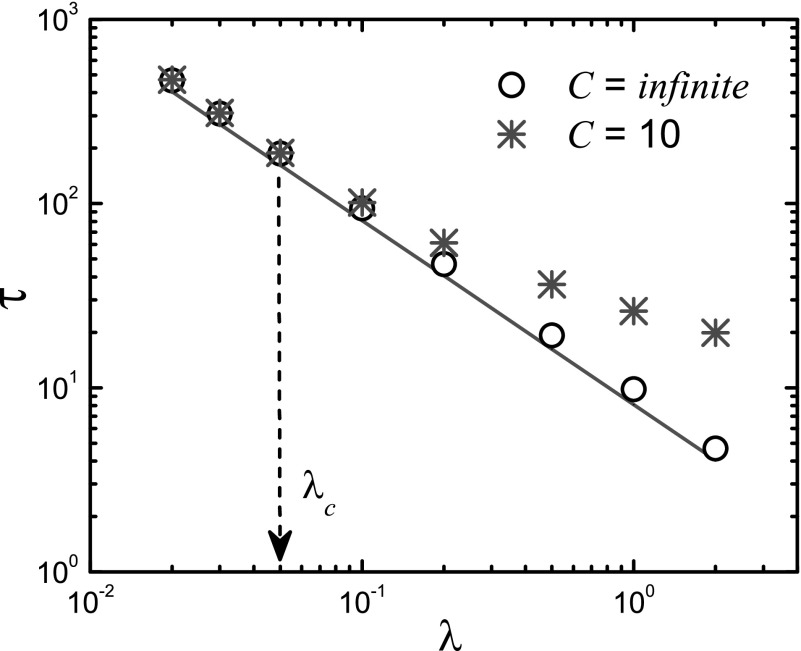
For the two cases where the capacity parameter *C* is infinite and finite (*C* = 10), the characteristic time τ as a function of the packet-generation rate λ. Other parameters are 〈k〉=10 and β=0.01. For *C* = 10, the critical packet-generating rate is $\lambda_{c}≃0.05$. Line represents the theoretical prediction [Eq. (6)].

We now turn to the more realistic case where the node delivering capacity is finite. The main difference from the infinite-capacity case lies in the likelihood of the emergence of traffic congestion in the network, which occurs when the packet-generating rate exceeds a critical value $\lambda_{c}$.^24^ Due to the complication associated with traffic congestion, our analysis for the infinite-capacity (or congestion-free) case cannot be applied, and we thus rely on numerical computation to explore the behavior of the characteristic time τ. Figure 4 shows the density of the infected nodes *i*(*t*) as a function of time *t* for *C* = 10. For comparison, we also include the case of C→∞. The critical value of $\lambda_{c}$ is determined to be about 0.05, and we set $\lambda =1>\lambda_{c}$ so that traffic congestion can occur. Figure 4 demonstrates that *i*(*t*) increases more slowly in the case of finite *C* than that of infinite *C*, indicating that traffic congestion can suppress the speed of epidemic spreading. The inset of Fig. 4 shows that, initially, the density of the infected nodes also increases exponentially with time for the finite-capacity case so that a characteristic outbreak time τ can still be defined. Figure 5 shows τ as a function of the packet-generation rate λ for both cases. We observe that τ scales inversely with λ for infinite *C*, as predicted by Eq. (6). For finite values of *C*, when free traffic flow is guaranteed $(\lambda <\lambda_{c}),\;\tau$ essentially exhibits the same behavior. However, for $\lambda >\lambda_{c}$ so that congestion can occur, the value of τ becomes larger, signifying a slowdown in the epidemic outbreak.

## IV. CONCLUSION

We have investigated the issue of the characteristic time associated with the outbreak of traffic-driven epidemic spreading on scale-free networks. Using physical reasoning, we have arrived at a formula relating the characteristic time to parameters underlying both the traffic and spreading dynamics, and also the ratio of the first to the second moment of the network betweenness distribution. It is found that traffic-flow condition plays an important role in the outbreak of epidemic spreading. Large-scale outbreak occurs more quickly as the packet-generation rate associated with the traffic dynamics is increased. However, an increase in the average network connectivity and the emergence of traffic congestion can slow down the epidemic outbreak, where the former shortens the average traveling time of a packet and decreases the number of packages passing through each node, resulting in a decrease in the infection probability of each node. In the presence of traffic jam, due to the fact that the number of packets in the queue of a jammed node exceeds its delivery capacity, the number of packets that can be forwarded is reduced compared to the case of absence of congestion. In this perspective, the emergence of congestion can effectively slow down the process of epidemic spreading.
